# Visceral fat obesity is highly associated with primary gout in a metabolically obese but normal weighted population: a case control study

**DOI:** 10.1186/s13075-015-0593-6

**Published:** 2015-03-24

**Authors:** Jennifer Lee, Ji-Yeon Lee, Jae-Ho Lee, Seung-Min Jung, Young Sun Suh, Jung-Hee Koh, Seung-Ki Kwok, Ji Hyeon Ju, Kyung-Su Park, Sung-Hwan Park

**Affiliations:** Division of Rheumatology, Department of Internal Medicine, School of Medicine, The Catholic University of Korea, Seoul St. Mary’s Hospital, 222 Banpo-daero, Seocho-gu, Seoul, 137-701 Republic of Korea; Department of Internal Medicine and Institute of Health Science, Gyeongsang National University School of Medicine, 79 Gangnam-ro, Jinju, 660-702 Republic of Korea; Division of Rheumatology, Department of Internal Medicine, School of Medicine, The Catholic University of Korea, St. Vincent’s Hospital, 93 Jungbu-daero (Ji-dong), Paldal-gu, Suwon, Gyeonggi-do 442-723 Republic of Korea

## Abstract

**Introduction:**

Gout is a chronic inflammatory disease the development of which is associated with obesity-induced metabolic abnormalities. However, a substantial number of non-obese patients (body mass index [BMI] <25 kg/m^2^) also develop gout in Korea. It was suggested that accumulation of visceral fat rather than subcutaneous fat is associated with metabolic abnormalities and hyperuricemia in patients with gout; therefore, we hypothesized that visceral fat accumulation was increased in non-obese gout patients.

**Methods:**

One hundred and three male patients with primary gout and 204 age-matched healthy controls who attended a health check-up examination were recruited after the review of medical charts. The visceral fat area (VFA) was measured using the bioelectrical impedance analysis (BIA) method, and a VFA >100 cm^2^ was defined as visceral fat obesity (VFO). The frequency of VFO was compared in patients and control groups. The frequencies of metabolic syndrome and related parameters were also investigated.

**Results:**

BMI, waist circumference, total fat mass, serum triglycerides, and serum glucose levels were significantly greater in patients compared with controls. VFA and the prevalence of VFO was increased in gout patients compared with controls. There were positive correlations between VFA and serum triglyceride levels and serum glucose levels. Multivariate regression analysis revealed that VFO is an independent risk factor for gout (odds ratio 2.488, 95% confidence interval 1.041–4.435). In non-obese subgroup analyses (gout patients, *n* = 38; healthy controls, *n* = 150), VFA (98.7 ± 19.3 vs. 91.0 ± 16.7, *P* = 0.016) and the frequency of VFO (47.4 vs. 27.3%, *P* = 0.017) remained significantly higher in gout patients. There was no difference in either BMI or total fat mass between patients and controls in the non-obese subgroup. The prevalence of metabolic syndrome in patients with gout was 31.7% (33/104), compared with 13.2% (5/38) in the non-obese subgroup according to modified ATP III criteria.

**Conclusion:**

VFO, measured using BIA, is observed more frequently in patients with primary gout compared with healthy controls, even in non-obese individuals. Therefore, VFO might more properly represent metabolic derangements in patients with gout than general obesity.

## Introduction

Gout is a prototypic of crystal-induced arthropathy caused by monosodium urate (MSU) crystal precipitation in joints. Although the precise mechanism of disease pathogenesis remains unclear, hyperuricemia is a prerequisite of gout development. In addition, the inflammasome, intracellular machinery related to innate immunity, plays an important role in producing IL-1β, which is the critical cytokine for MSU-induced gout inflammation [1]. The history of gout spans thousands of years, and it was historically referred to as a king’s disease because its development is closely associated with the consumption of large amounts of fatty foods and alcohol, as well as obesity. Indeed, several studies reported a close relationship between fat accumulation and gout/hyperuricemia [2-6]. Furthermore, gout patients are not only obese but also commonly have obesity-associated comorbidities such as high blood pressure, hypertriglyceridemia, or impaired fasting glucose, which together comprise metabolic syndrome [7]. In line with this, the prevalence of metabolic syndrome is higher in gout patients compared with the general population [8-14].

These observations suggest that obesity is a strong risk factor for the development of gout. However, in Korea, there are a substantial number of non-obese gout patients (body mass index (BMI) <25 kg/m^2^). It was suggested that the accumulation of visceral fat rather than subcutaneous fat is associated with metabolic abnormalities and hyperuricemia in patients with gout [4]; therefore, we hypothesized that visceral fat accumulation was increased in non-obese gout patients and that this resulted in metabolic derangements that caused these individuals to be more prone to gout. In the present study, we investigated the association between visceral fat obesity and the development of gout, focusing particularly on a non-obese population, and characterized metabolic syndrome-related parameters in obese and non-obese subgroups of gout patients.

## Methods

### Study population

One hundred and three patients who were diagnosed with gout according to the 1977 American College of Rheumatology preliminary criteria for gout [15] were recruited between March 2009 and Dec 2013 at Seoul St Mary’s hospital, Seoul, Korea. Two hundred and four age-matched healthy individuals who attended routine health check-up examinations during the same period were included as controls. Retrospective medical chart reviews were performed to obtain clinical and laboratory data; thereby, no consent was needed from the study population. The Institutional Review Board of the School of Medicine, Catholic University, approved the study protocol.

### Clinical information and laboratory analysis

The clinical information was obtained from electronic medical records of all patients, including medical history, the use of medications, and laboratory data. Anthropometric variables (height and weight) were measured when subjects were in the standing position. BMI was calculated as weight in kg divided by the square of height in meters (kg/m^2^). Blood pressure was measured in the sitting position (in mmHg). The visceral fat area (VFA) was determined using the bioelectrical impedance analysis (BIA) method with Inbody 720 (Biospace, Seoul, Korea) according to the manufacturer’s instructions. Fasting venous blood samples were taken to measure the serum levels of total cholesterol, high-density lipoprotein (HDL) cholesterol, low-density lipoprotein (LDL) cholesterol, triglycerides, glucose, and uric acid.

### Definitions

General obesity was defined as a BMI >25 kg/m^2^ because the study population was Asian. Visceral fat obesity (VFO) was defined as a VFA >100 cm^2^ according to a previous report [5]. The presence of metabolic syndrome was defined according to the modified ATP (Adult Treatment Panel) III criteria, in which the waist circumference criterion was adjusted to ≥90 cm in males and ≥80 cm in females according to the World Health Organization (WHO) Asia-Pacific obesity criteria (APC) [16]. All other components were the same as in the ATP III criteria [17], where the presence of metabolic syndrome was defined as having at least three of the following five parameters: a waist circumference >102 cm for males or >88 cm for females, serum triglyceride levels ≥150 mg/dL, serum HDL cholesterol levels <40 mg/dL for males or <50 mg/dL for females, systolic blood pressure ≥130 mmHg or diastolic blood pressure ≥85 mmHg, and fasting blood glucose ≥110 mg/dL.

### Statistics

Statistical analyses were performed using the SAS software, version 9.0 (SAS, Cary, NC, USA), and data are presented as mean ± standard deviation or median (interquartile range). Data were compared using the Student’s *t*-test or the Mann-Whitney *U*-test for continuous variables, and the Chi-square test for categorical variables. Spearman’s correlation coefficient was calculated for correlation analysis. Multivariate logistic regression analysis was used to identify independent risk factors for the development of gout, and the results are presented as odds ratio (OR) with 95% CI. A value of *P* <0.05 was used to indicate statistical significance.

## Results

### Clinical and laboratory characteristics of the subjects

A total of 104 gout patients and 203 age-matched healthy controls were recruited. Table 1 shows the clinical and laboratory characteristics of the subjects. All the subjects were male, and the mean age was comparable between groups (51.0 (16) years for gout patients versus 51.0 (14) years for healthy controls). Among 104 gout patients, 72 were taking a urate-lowering agent at the time of body composition analysis; 28 patients were taking anti-hypertensive medication, and 11 were on a glucose-lowering agent due to diabetes mellitus, and 39 patients were taking a lipid-lowering agent such as statins or omega-3 fatty acids. Gout patients had a significantly higher mean BMI (25.8 (4.6) versus 23.5 (3.1) kg/m^2^, *P* <0.001), VFA, waist/hip circumference, total fat mass and percent mass, blood pressure, serum uric acid levels, serum triglycerides, and glucose levels. In particular, the frequency of VFO was higher in gout patients (71.8 versus 41.2%, *P* <0.001). There were no differences in the serum level of total cholesterol or LDL cholesterol between the two groups.

**Table 1:** **Clinical and laboratory characteristics of the subjects**

	**Gout**	**Healthy**	***P***
	**(n = 103)**	**(n = 204)**	
Age, years	51.0 (16)	51.0 (14)	0.944*
Body mass index, kg/m^2^	25.8 (4.6)	23.5 (3.1)	<0.001*
Visceral fat area, cm^2^	115.6 ± 25.3	97.7 ± 20.2	<0.001
Visceral fat obesity, VFA ≥100 cm^2^	74 (71.8%)	84 (41.2%)	<0.001
Waist circumference, cm	91.2 ± 9.7	82.3 ± 7.5	<0.001
Hip circumference, cm	99.2 ± 7.4	94.0 ± 4.5	<0.001
Total fat mass, kg	20.1 (10.3)	14.9 (5.4)	<0.001*
Total fat percentage, %	25.6 ± 5.7	21.7 ± 4.4	<0.001
Systolic blood pressure, mmHg	125.0 ± 13.8	118.5 ± 12.1	<0.001
Diastolic blood pressure, mmHg	80.4 ± 11.1	75.7 ± 9.9	0.001
Uric acid, mg/dL	7.9 ± 1.9	6.4 ± 6.0	0.001
Total cholesterol, mg/dL	191.2 ± 37.0	192.0 ± 27.2	0.857
Triglyceride, mg/dL	155.0 (118)	82.0 (52.0)	<0.001*
High-density lipoprotein, mg/dL	44.0 (15.0)	51.0 (15.0)	<0.001*
Low-density lipoprotein, mg/dL	114.3 ± 32.5	119.1 ± 25.5	0.159
Glucose, mg/dL	96.4 (16.0)	89.0 (10.0)	<0.001*

*Non-parametric Mann-Whitney *U*-test was used.

mean ± standard deviation or median (interquartile range).

### Visceral fat obesity is an independent risk factor for the development of gout

**Figure 1:**
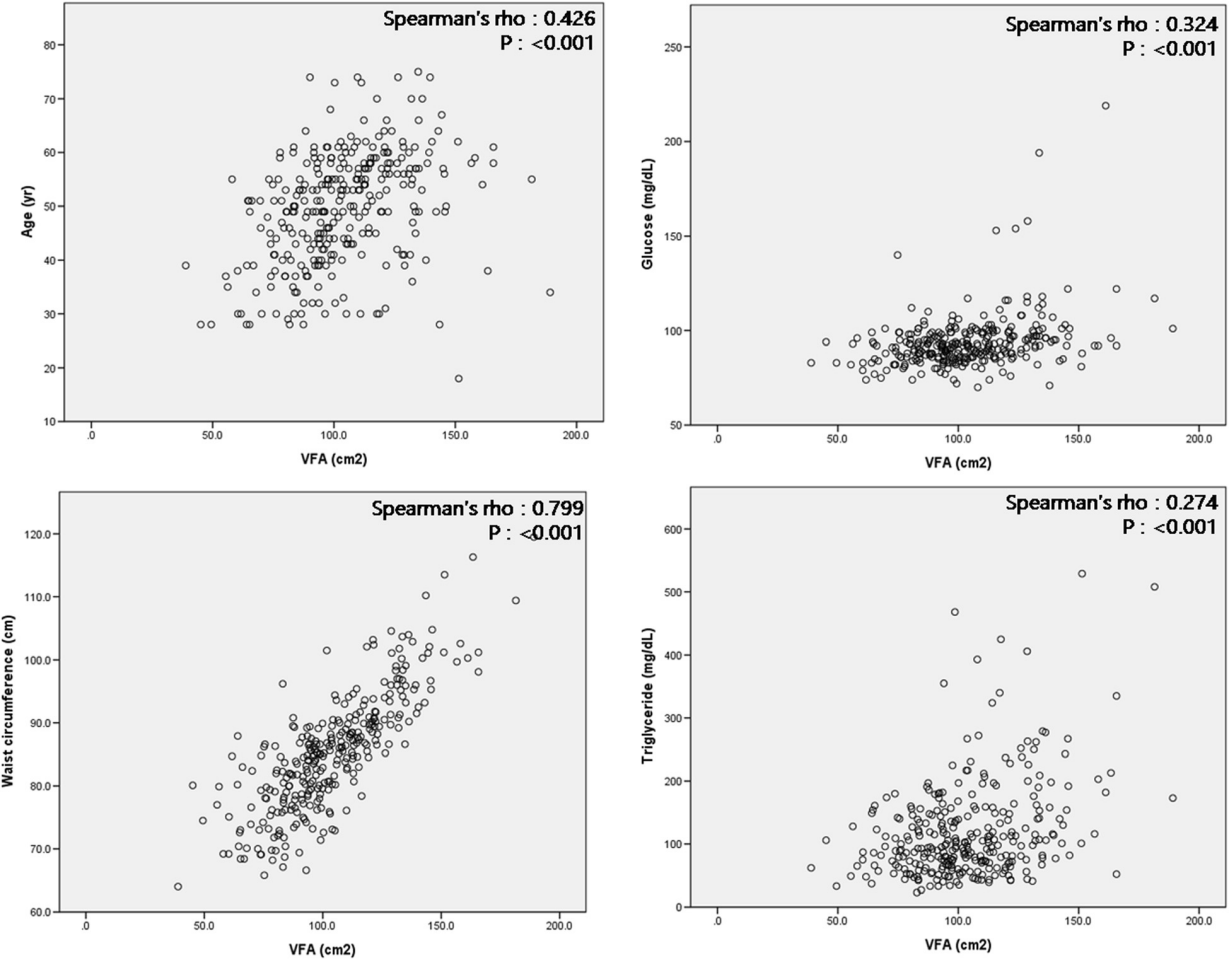
**Correlation between visceral fat area (VFA) and metabolic syndrome-related parameters.** Spearman’s correlation between visceral fat area and age, serum glucose level, waist circumference, and serum triglyceride level.

VFA was correlated positively with age (Spearman’s rho: 0.426, *P* <0.001) and other metabolic syndrome-associated factors including waist circumference (Spearman’s rho: 0.799, *P* <0.001), serum fasting glucose levels (Spearman’s rho: 0.324, *P* <0.001) and triglycerides (Spearman’s rho: 0.274, *P* <0.001) (Figure 1), suggesting that VFA reflects metabolic factors accurately. Furthermore, because we observed that the frequency of VFO was significantly higher in gout patients compared with controls, we next investigated whether VFO was an independent risk factor for gout. Multivariate logistic regression analysis of variables including age, the presence of obesity (BMI ≥25 versus BMI <25 kg/m^2^), the presence of hypertriglyceridemia (≥150 mg/dL versus <150 mg/dL), and the presence of VFO revealed that VFO was an independent risk factor for gout development (OR 2.488, 95% CI 1.041, 4.435) (Table 2). Obesity was also an independent risk factor, consistent with previous reports that reported a close association between obesity and gout [18,19].

**Table 2:** **Multivariate logistic regression analysis for the development of gout**

	**β**	**Standard error**	**Odds ratio**	**95% CI**
Age	-0.009	0.016	0.991	0.961, 1.022
Body mass index ≥25 kg/m^2^	0.911	0.773	2.488	1.298, 4.770
Visceral fat area ≥100 cm^2^	0.765	0.37	2.149	1.041, 4.435
Triglyceride ≥150 mg/dL	2.007	0.32	7.442	3.972, 13.943

### Metabolic derangements in non-obese gout patients

Subgroup analyses were performed to investigate whether metabolic derangements were present in both obese and non-obese gout patients. A total of 38 of the 104 gout patients (36.5%) had BMI <25 kg/m^2^, and their clinical and laboratory characteristics were compared with those of non-obese healthy controls (n = 150/203, 69.0%) (Table 3). Although BMI was comparable between groups (23.4 (2.0) kg/m^2^ for gout patients versus 22.7 (2.4) kg/m^2^ for healthy controls, *P* = 0.127), VFA (98.7 ± 19.3 versus 91.0 ± 16.7 cm^2^, *P* = 0.016) and the frequency of VFO (47.4 versus 27.3%, *P* = 0.017) were higher in gout patients. Non-obese gout patients also had significantly higher serum triglyceride (127.0 (94.0) versus 81.0 (52.0) mg/dL, *P* <0.001) and glucose (97.3 ± 13.0 versus 89.0 ± 6.8 mg/dL, *P* <0.001) levels compared with non-obese normal individuals. Of note, total fat mass (14.3 (4.5) versus 13.5 (4.1) kg, *P* = 0.271) and the total fat percentage (21.1 ± 4.7 versus 20.4 ± 3.8%, *P* = 0.295) were not different between the two subgroups. Collectively, these results suggest that metabolic derangements occur in non-obese gout patients, and that visceral fat accumulation rather than the amount of total fat is associated with this phenomenon.

**Table 3:** **Clinical and laboratory characteristics of the non-obese subjects (BMI <25 kg/m** ^**2**^ **)**

	**Gout**	**Healthy**	***P***
	**(n = 38)**	**(n = 150)**	
Age, years	52.6 ± 9.7	49.2 ± 10.8	0.06
Body mass index, kg/m^2^	23.4 (2.0)	22.7 (2.4)	0.127*
Visceral fat area, cm^2^	98.7 ± 19.3	91.0 ± 16.7	0.016
Visceral fat obesity, VFA ≥100 cm^2^	18 (47.4%)	41 (27.3%)	0.017
Waist circumference, cm	84.7 (6.8)	79.7 (8.5)	0.001*
Hip circumference, cm	92.9 ± 4.9	92.4 ± 3.7	0.403
Total fat mass, kg	14.3 (4.5)	13.5 (4.1)	0.271*
Total fat percentage, %	21.1 ± 4.7	20.4 ± 3.8	0.295
Systolic blood pressure, mmHg	122.5 ± 13.9	117.5 ± 13.1	0.049
Diastolic blood pressure, mmHg	80.0 (14.5)	74.0 (15.0)	0.070*
Uric acid, mg/dL	7.9 (1.3)	6.0 (1.3)	<0.001*
Total cholesterol, mg/dL	193.1 ± 33.8	190.5 ± 27.5	0.746
Triglyceride, mg/dL	127.0 (94.0)	81.0 (52.0)	<0.001*
High-density lipoprotein, mg/dL	46.5 (16.0)	51.0 (15.0)	0.008*
Low-density lipoprotein, mg/dL	114.5 (37.0)	115.0 (37.0)	0.919*
Glucose, mg/dL	97.3 ± 13.0	89.0 ± 6.8	<0.001

*Non-parametric Mann-Whitney *U*-test was used.

mean ± standard deviation or median (interquartile range).

### Prevalence of metabolic syndrome and the fulfillment of related parameters

The prevalence of metabolic syndrome in gout patients was 31.7% (33/104) according to the modified ATP III criteria. Table 4 shows which components of metabolic syndrome were present in the current study subjects. In non-obese subgroup analyses, the prevalence of metabolic syndrome was 13.2%. Interestingly, only one patient (1/38, 2.6%) had a waist circumference ≥90 cm, whereas the frequency of VFO was 47.4% (18/38). The frequency of hypertriglyceridemia, elevated blood pressure, and impaired fasting glucose was significantly higher in gout patients.

**Table 4:** **Prevalence of the fulfillment of metabolic syndrome-related parameters**

	**Body mass index ≥25**			**Body mass index <25**		
	**Gout**	**Healthy**	***P***	**Gout**	**Healthy**	***P***
	**(n = 65)**	**(n = 54)**		**(n = 38)**	**(n = 150)**	
WC ≥90 cm	50 (76.9%)	24 (44.4%)	<0.001	1 (2.6%)	5 (3.3%)	0.826
TG ≥150 mg/dL	41 (63.1%)	6 (11.1%)	<0.001	13 (34.2%)	15 (10.0%)	<0.001
HDL <40 mg/dL	28 (43.1%)	8 (14.8%)	0.001	10 (26.3%)	14 (9.3%)	0.011
BP ≥130/85	31 (47.7%)	15 (27.8%)	0.026	15 (39.5%)	26 (17.3%)	0.003
Glucose ≥110 mg/dL	15 (23.1%)	0 (0%)	<0.001	5 (13.2%)	0 (0%)	<0.001
modified ATP III	28 (43.1%)	1 (1.9%)	<0.001	5 (13.2%)	0 (0%)	<0.001

Results are presented as number (%) of patients. BP: blood pressure; HDL: high density lipoprotein; TG: triglyceride; WC: waist circumference.

## Discussion

In the present study, we demonstrated that VFA, measured using BIA, was greater in gout patients compared with healthy controls. VFO was more prevalent in non-obese gout patients compared with non-obese controls. Furthermore, metabolic derangements according to the components of metabolic syndrome were also present in non-obese gout patients.

Consistent with previous studies suggesting that adipose tissue plays an important role in gout [4,9,12-14,20], the current data also demonstrated that obesity is an independent risk factor for gout. In particular, the accumulation of visceral fat was detrimental, even in patients without general obesity. Although the precise molecular mechanism behind this phenomenon remains unclear, the contribution of fat accumulation to the development of gout has two possible explanations. First is the traditional correlation between fat accumulation and hyperuricemia. It has been reported that fat affects uric acid metabolism and results in increased serum uric acid levels [2,3,20,21]. However, we did not identify any associations between the fat accumulation and serum uric acid levels. This discrepancy might be because most of the patients were taking uric acid-lowering agents as well as the relatively small sample size of the study. The second explanation is that the production of adipokines by fat tissue, such as IL-6 and tumor necrosis factor-α, enhances obesity-induced inflammation [22]. Importantly, these adipokines that promote inflammation are mainly produced by visceral rather than subcutaneous fat [23]. On the contrary, the level of adiponectin, an anti-inflammatory adipokine, is lower in individuals with VFO [24]. This adipokine profile supports the emphasis on VFO in the current study.

Recent studies suggested another possible explanation involving activation of the NLRP3 inflammasome and obesity-induced inflammation [15]. Triglycerides, the level of which was highly associated with VFO, are the source of fatty acids that stimulate toll-like receptor (TLR)-2 and can subsequently activate the inflammasome, which plays a central role in acute gout attacks [25]. In turn, the inflammasome enhances fat accumulation [26]. This suggests that this vicious cycle might be present in gout patients in whom the inflammasome is activated, although future studies are needed to confirm this hypothesis.

The current study demonstrated that VFO was more prevalent in non-obese gout patients with BMI <25 kg/m^2^ compared with non-obese control subjects. Importantly, there was no difference in total fat mass or total fat percentage between gout patients and controls. Therefore, these data suggest that visceral obesity, regardless of general obesity, contributes to the pathogenesis of gout. In addition, non-obese gout patients had hypertriglyceridemia and were pre-hypertensive more frequently than healthy controls. Because the waist circumference of most individuals did not exceed 90 cm, while >25% of individuals exhibited VFO, visceral fat accumulation appears to represent central obesity and metabolic abnormality more accurately in this population. As the BIA method is a safer and more convenient method for measuring VFA compared with traditional computed tomography (CT), it might be helpful for assessing and monitoring visceral fat accumulation in gout patients.

One interesting question is whether the presence of VFO affects the clinical manifestation of gout. For example, Gheita *et al*. demonstrated that bone erosion was more prevalent in gout patients with metabolic syndrome compared with those without metabolic syndrome [27]. However, there were no differences in the frequency of bone erosion at the affected joints in individuals with and without VFO in the current study (data not shown).

We verified that the prevalence of metabolic syndrome was higher in gout patients, which was consistent with previous studies [8,10,12-14]. The proinflammatory trait of the patients with metabolic syndrome suggested by Zhao *et al*. [28] who demonstrated that the level of highly sensitive C-reactive protein, an acute phase reactant, was higher in individuals with metabolic syndrome may contribute to gouty inflammation. Importantly, the current data revealed that a number of non-obese gout patients fulfilled the criteria for metabolic syndrome, albeit to a lesser extent than did obese patients. As mentioned above, most of these patients had a waist circumference <90 cm. Therefore, other components of metabolic syndrome are likely to be associated with VFO.

The current study has several limitations. First, measuring visceral fat using BIA might not be the gold-standard method. However, the device we used in this study was validated in comparisons with CT scans [29] and the accuracy of the BIA method is well-established [30-32]. Second, this is a retrospective study based on the review of medical charts, and had a relatively small sample size. However, an important strength of the current study is that we verified that visceral fat accumulation was present and represented metabolic abnormalities in non-obese gout patients. Therefore, this suggests that assessing and monitoring VFO might be required.

## Conclusions

VFO, as measured using BIA, was observed more frequently in both obese and non-obese patients with primary gout compared with healthy controls. VFA was correlated positively with components of metabolic syndrome. Therefore, VFO might more properly represent metabolic derangements in patients with gout than general obesity. Future studies addressing whether the risk of gout can be reduced by monitoring and losing visceral fat will be promising.

## Abbreviations

APC: Asia-pacific obesity criteria
ATP: Adult Treatment Panel
BIA: bioelectrical impedance analysis
BMI: body mass index
HDL: high-density lipoprotein
IL: interleukin
LDL: low-density lipoprotein
MSU: monosodium urate
OR: odds ratio
TLR: Toll-like receptor
VFA: visceral fat area
VFO: visceral fat obesity
